# Source plasma collection in the United States: Toward a more personalized approach

**DOI:** 10.1002/ajh.25817

**Published:** 2020-04-20

**Authors:** Jan Hartmann, Michael J. Ragusa, Mark A. Popovsky, Susan F. Leitman

**Affiliations:** ^1^ Haemonetics Corporation Boston MA USA; ^2^ NIH Clinical Center National Institutes of Health Bethesda MD USA

To the Editor:

Donor plasmapheresis is one of the most frequent medical procedures performed in healthy individuals in the United States (U.S.). In 2017 alone, approximately 30 million liters of source plasma were collected in approximately 40 million procedures in the U.S.

Donated plasma is integral to satisfying the growing need for plasma‐based medicines. This increasing need is driven by demographic changes, epidemiology, improvements in diagnostic rates, and new and expanding therapeutic uses. Key indications include inherited and acquired immune deficiency disorders and autoimmune disorders.

The majority of the global source plasma supply is collected in the U.S., where donors are compensated and federal regulations permit more frequent and higher volume donations than in Europe. The U.S. is self‐sufficient for source plasma, a goal that many European countries still aim to achieve. However, there currently is a critical shortage of plasma‐derived medicines, particularly of intravenous immunoglobulin (IVIG)^1^ in the U.S. and abroad that is impacting patients.

Concerns for donor safety focus on well‐understood short‐term effects, most importantly, vasovagal hypotensive events and, less frequently, citrate reactions. Longer‐term potential complications, including iron or protein depletion and osteoporosis, are less well‐characterized. Previous studies have demonstrated a temporary reduction in serum protein levels but have also shown a rebound effect, even with intensive donation schedules.^2^ Regular monitoring of protein levels in serial plasma donors has been incorporated into federal regulations as a safety measure (21CFR630.15 and 21CFR640.65). The hypothesized risks of osteoporosis due to citrate effects and iron depletion have not been confirmed and long‐term observational studies have established the safety of regular donations.^3^

A nomogram regulating the volume of source plasma that can be extracted per donation serves as a key instrument to ensure donor safety and, in particular, to reduce the likelihood of hypotensive events. The U.S. Food and Drug Administration (FDA) issued its current plasmapheresis nomogram in 1992.^4^ To maximize ease of use and to minimize operator error, the nomogram consists simply of three allowable collection volume categories based on donor weight.

For nearly 30 years, this nomogram has proven to be effective, resulting in a strong safety record for donor plasmapheresis. Reaction rates are very low, with fewer than 0.03% severe reactions.^5^ However, while the 1992 nomogram fulfilled the objective of simplicity, it has limitations. It does not account for height or body mass index (BMI), nor for hematocrit levels. These factors are known to influence the total plasma volume (TPV) of a donor and would be valuable components of a more personalized approach to calculate target collection volumes.^6^ Moreover, the current weight‐based step‐wise approach leads to abrupt target changes of up to 20% between groups. In summary, while the safety profile has been good across the donor population, there could be sub‐groups of donors at increased, yet currently unquantified risk.

Here, we report a systematic retrospective analysis of a large real‐world data set of source plasma collections following current U.S. standards to better understand the implications and potential opportunities for improvement. De‐identified data from 111 916 routine plasma collections (all‐comers) performed in February 2019 at 86 nationwide U.S. plasma donation centers (Octapharma Plasma, Charlotte, North Carolina) were obtained. For each donation, donor weight, height and hematocrit level, as well as the plasma volume collected (PVC), which was derived from the target plasma volume, were documented. Routine donor‐specific parameters were analyzed. The TPV was calculated by first estimating each donor's BMI‐adjusted blood volume per kilogram, and then factoring in the donor weight and hematocrit level.^7^ The PVC was then compared to the TPV for each donation. Mean plasma volume collected was 760 mL, with highest plasma yields in the high‐weight group (>175 lb) and the lowest yields in the low‐weight group (110‐149 pounds). A substantial majority (73%) of the donors were in the highest weight category.

An analysis of the amount of PVC as a percentage of the donor's TPV (PVC/TPV), when plotted against the TPV, showed a distribution along three bands, representing the three weight categories (Figure 1A). This pattern is in accordance with the expected theoretical patterns. However, the distribution along the bands offered additional insights into the heterogeneity of these values in a real‐world data set.

**FIGURE 1:**
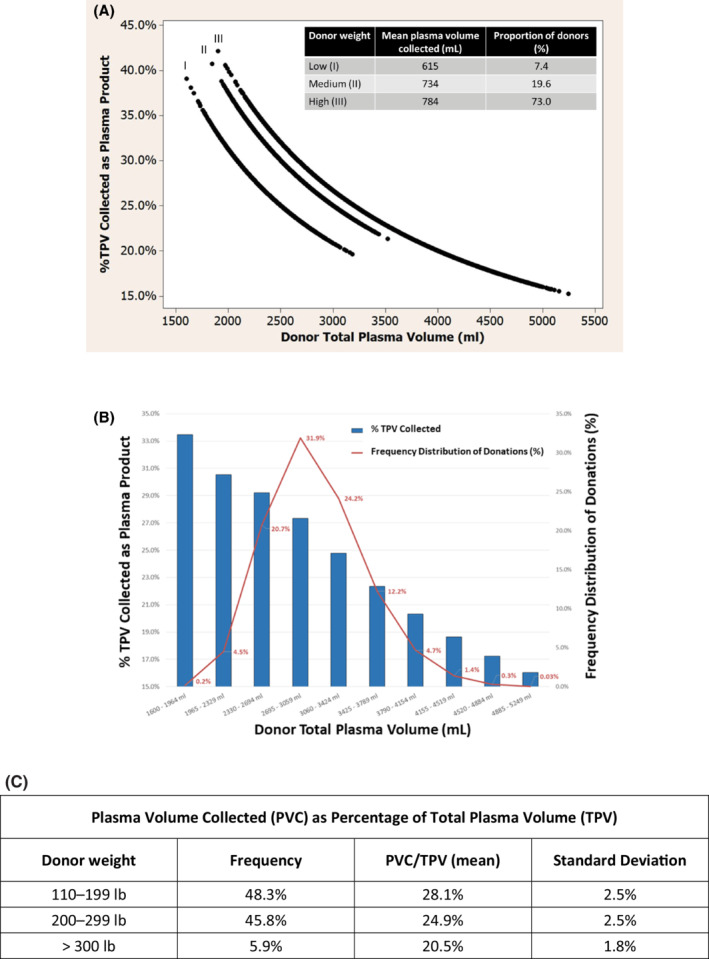
A, Distribution of the percentage of the donor's plasma volume collected in the plasma product as a function of the donor's total plasma volume (TPV). Three discrete bands are described by the three different nomogram weight groups. B, Distribution of donors and the percent contribution of plasma across the total plasma volume. C, Plasma volume collected as a percentage of total plasma volume for adjusted weight categories [Color figure can be viewed at wileyonlinelibrary.com]

The PVC/TPV values ranged from 15%‐42% between all individuals. The ratio of PVC to TPV was inversely proportional to weight, with a mean of 28.1% in donors weighing 110‐199 pounds, 24.9% in donors weighing 200‐299 pounds, and 20.5% in donors weighing 300‐400 pounds (Figure 1C). Similarly, the described bands showed an inversely proportional distribution where the highest PVC/TPV values were observed in donors with lowest TPV. When analyzed by discrete TPV categories, the inverse trend was confirmed (Figure 1B). The highest percentage collection volume occurred in donors with the lowest TPV. This analysis more clearly demonstrates the distribution of donors and the percent contribution across the TPV spectrum. The majority (76.8%) of donations were performed in donors whose TPV ranged from 2330‐3424 mL. In these donors, the percentage of TPV donated averaged 27%. However, the 4.7% of donations in lower TPV donors (1600‐2329 mL TPV) yielded on average a plasma collection volume that was 31% of TPV, while the highest TPV donors (>3425 mL) averaged a collection volume that was 22% of TPV.

The need for plasma‐derived medicines is increasing. Recent issues with drug availability highlight the vulnerability of the supply chain and the impact on patient treatment. The plasma collection nomogram issued by the FDA in 1992 is limited to only three weight‐based categories. To our knowledge, this is the first systematic, although retrospective, analysis of a large real‐world data set that explores inter‐individual differences in absolute and relative plasma collection volumes following this nomogram.

Our findings demonstrate a very large range in plasma volumes collected as a proportion of total plasma volume, varying by more than a factor of two between individual donors. Paradoxically, the highest relative donation volumes were observed in donors with the lowest total plasma volumes.

Our analysis raises two key questions. First, the widely discrepant percentage of plasma volume collected suggests that significant differences exist in individual donor risk exposure, despite a good overall population‐wide safety record. Although the existing literature on plasmapheresis addresses both short‐ and long‐term adverse donor events and attempts to identify individual risk factors, it does not address whether it can be considered safe to allow some donors to donate more than twice the percentage of their total plasma volume than others. Second, the efficiency of the source plasma collection system seems sub‐optimal, wherein some donors are only permitted to contribute a relatively small percentage of their total plasma volume. This is particularly critical given the recent supply issues and the fact that the U.S. is the major contributor to the global supply of plasma‐derived medicines.

Limitations of our analysis include the lack of clinical outcomes data, such as donor adverse events. Future studies using actual collection volumes should include analyses of donor outcomes. Our data was obtained from 86 plasma collection centers by one commercial plasma collector (Octapharma Plasma). The time period was randomly selected and the 86 centers are representative of the overall donor population of this plasma collector. However, it is worth considering that there may be slight differences between the populations of different plasma collectors and that there are seasonal effects on plasma collection frequencies and adverse event rates, although neither of these would influence target volumes.

The 1992 nomogram has served plasma donors well and kept them collectively safe; the event rates for moderate and severe hypotensive events are very low. However, the use of an exclusively weight‐based approach along three weight categories has led to a skewed practice, where a disproportionately high volume of plasma is collected relative to available total plasma volume in smaller donors. Conversely, an inefficiently low volume of source plasma is collected, relative to total plasma volume, in larger persons.

Further studies are needed to better understand the risks associated with high PVC/TPV values, and to explore the benefits of a more personalized approach. This might include the creation of a continuous algorithm for individual targeted donation volumes tailored to the donor's TPV. We hypothesize that this type of approach would improve the overall benefit‐to‐risk profile of donor plasmapheresis, while further supporting individual donor safety. The simplicity of the 1992 nomogram reflected the desire to reduce the risk of human error during a manual, labor‐intensive collection process. With improved technology and collection systems capable of managing multifactor‐targeted settings, these risks have been greatly reduced. This should enable the use of a new, more personalized approach, provided the current safety profile is preserved or even improved.

Large‐scale, multicenter, prospective, randomized, controlled clinical trials are needed to demonstrate the benefits and safety of such approach. In times of personalized medicine, we hope that our findings can contribute to a discussion about a paradigm shift toward a more personalized collection approach that would improve the individual safety profile for donors, while potentially supporting healthcare systems that have a pressing need for additional source plasma.

## CONFLICT OF INTERESTS

Dr. Hartmann and Michael Ragusa are employees of Haemonetics Corporation; Dr. Popovsky serves as safety consultant to Haemonetics Corporation and receives reimbursement for his work; Dr. Leitman has served as a consultant to, and participated in advisory committee meetings for, Haemonetics Corporation and has received reimbursement for her work.
